# Rift Valley fever seroprevalence and abortion frequency among livestock of Kisoro district, South Western Uganda (2016): a prerequisite for zoonotic infection

**DOI:** 10.1186/s12917-018-1596-8

**Published:** 2018-09-03

**Authors:** Ngabo Herbert Budasha, Jean-Paul Gonzalez, Tesfaalem Tekleghiorghis Sebhatu, Ezama Arnold

**Affiliations:** 1 Kisoro District Local Government, Department of Production and Marketing, Office of the District Veterinary Officer, P.O Box 123, Kisoro, Uganda; 2 Center of Excellence for Emerging & Zoonotic Animal Disease (CEEZAD) Kansas State University, Office Park, 1800 Kimball Ave, Suite 130, Manhattan, KS 66502 USA; 3 College of Veterinary Medicine, International Programs, Kansas State University, Office Park, 1800 Kimball Ave, Suite 180, Manhattan, KS 66502 USA; 4 Department of Health and Social services, Uganda Red Cross Society, P.O.Box 494, Kampala, Uganda

**Keywords:** Rift Valley fever, Seroprevalence, Livestock, Kisoro, Abortion

## Abstract

**Background:**

Rift Valley fever (RVF) is classified as viral hemorrhagic fever and is endemic in East and West Africa. RVF is caused by an arthropod borne virus (RVFV); the disease is zoonotic and affects human, animal health as well as international trade. In livestock it causes abortions, while human infection occurs through close contact with infected animals or animal products.

**Methods:**

A quantitative observational study using stratified sampling was conducted in the western region of Uganda. Blood samples and abortion events from 1000 livestock (goats, sheep and cattle) was collected and recorded. Serum was analyzed for RVFV IgG reacting antibodies using competitive ELISA test.

**Results:**

The overall RVFV seroprevalence was of 10.4% (104/1000). Cattle had the highest seroprevalence (7%) followed by Sheep (2.2%) then goats (1.2%). Species specific RVFV seroprevalence was highest in cattle (20.5%) followed by sheep (6.8%) then goats (3.6%). RVFV seroprevalence in northern highlands (21.8%) was significantly higher (*p* < 0.001) than in the southern lowlands (3.7%). Overall prevalence of abortion was (17.4%), sheep had the highest prevalence of abortion (7.8%) followed by goats (6.3%) and then cattle (3.3%). Species specific abortion prevalence was highest in Sheep (24.1%) followed by goats (18.8%) and then 9.7% in cattle.

**Conclusion:**

RVFV is endemic in Kisoro district and livestock in the highland areas are more likely to be exposed to RVFV infection compared to those in the southern lowlands. Out breaks in livestock most likely will lead to zoonotic infection in Kisoro district.

## Background

Rift Valley fever (RVF) is a zoonotic disease caused by Rift Valley Fever virus (RVFV), a member of the Phlebovirus genus in the *Phenuiviridae* family [1]. RVFV is transmitted by mosquitoes and primarily affects ruminants causing abortions in pregnant females, acute mortality in young susceptible livestock, and haemorrhagic fever [2, 3]. In humans, the majority of infections result from direct or indirect contact with the blood or tissues of infected animals [3]. Bites from infected mosquitoes cause infection in the humans [4, 5]. The disease in humans can be asymptomatic or presents with mild flu-like symptoms to severe illness with symptoms such as hepatitis, encephalitis and retinitis [6, 7]. Prevention of RVF involves identifying and isolation of sick animals to minimize infection to other animals as well as zoonotic spread, use of protective wear when dealing with tissues and blood from animals, sleeping under mosquito nets in endemic areas, proper handling and cooking of animal products before consumption. Vaccination of livestock against RVFV reduces infection and spread in cattle and therefore in human [8, 9]. RVF epidemics in East Africa and surrounding countries often happen after heavy and longer than usual rainy periods, that lead to proliferation of mosquito vectors of RVFV [10, 11].

RVF was first identified in the Rift valley of Kenya in 1930–31 while first human cases in Uganda were identified in 1968 [12–14]. Between 2006 and 2008, the East African countries of Kenya and Tanzania and other countries: Somalia, Sudan, Madagascar and South Africa had outbreaks [15–22]. From 1977 to 1993, outbreaks occurred in Egypt, Senegal and Mauritania [23–26]. Potential for spread to other continents was realized when Swedish soldiers from war in Egypt reacted positive to RVFV [27]. In 2000, RVF was identified off the African continent in Saudi Arabia and Yemen [28, 29]. Because of the ubiquity of its vectors and the continuous increase in international trade and exchanges, RVFV presents a constant global risk of expansion out of its original domains [30].

In 2012, the district veterinary office of Kisoro reported in the official monthly surveillance reports of May, 2012 that RVF-specific antibodies (IgG) were detected from one cow specimen from Nyakabande sub-county by surveillance team from the Ministry of Agriculture, Animal Industry and Fisheries (MAAIF). Recent RVF outbreaks in animals also caused human mortalities in Kabale district, Western Region of Uganda in 2016 [9]. For over two decades (1997–2015), complaints from residents, and from the official communication from the office of the District Veterinary Sector, abortion storms in domestic ungulates have often occurred during the rainy season of “*Gichurasi”* (March to May). Official communication from the District Health Officer indicated that humans too experienced more abortions around the same period. Important risk factors such as: livestock movement from cattle markets between Kabale, Kisoro and bordering countries known to be RVFV endemic like Rwanda [31], optimum weather conditions, ubiquity of mosquitoes, presence of wildlife and topographical variations create an increasing risk of emergence and spread of RVFV specifically in the Western Region of Uganda [5, 8, 22, 29, 32]. The aim of the study was to determine seroprevalence of RVFV in livestock and, express association between RVFV in livestock to abortion bouts in Kisoro district with the ultimate goal of increasing awareness to the presence of RVF in Kisoro district hence leading to improved surveillance to prevent, detect and respond to future infections and outbreaks in livestock that may also become zoonotic.

## Methods

### Study area

**Fig. 1:**
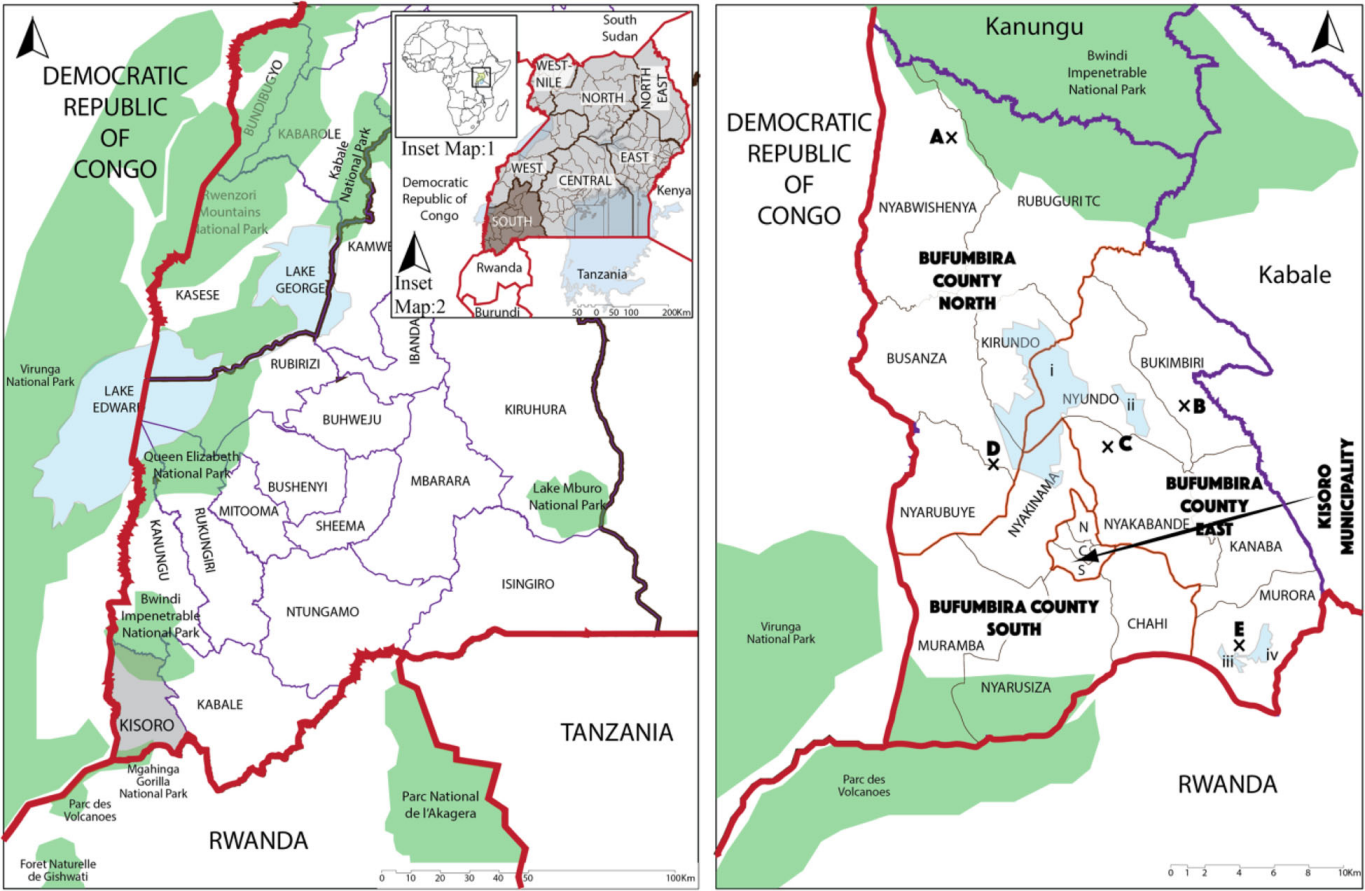
Study sites from the South Western province (Uganda, left panel) of the Kisoro District (Right panel) including the sub counties. Legend: Boundaries (lines): Country = Red; Region = Black; District = Purple; County = Orange. Cattle markets (X): A = Rubuguri; B = Kateretere; C = Rwivovo; D = Mupaka; E = Iryaruhuri. Water Bodies = shaded blue (lakes: i = Mutanda; ii = Mulehe; iii = Chahafi; iv = Kayumbu). Shaded green = National Parks. Fig. 1 was generated by the authors. It was generated for this research work.

Study was carried out in Kisoro district, Kigezi region, Western Region of Uganda (Fig. 1). The district has 14 Sub counties, with 34 parishes and a total of 390 villages [33]. The district borders Rwanda and Democratic Republic of Congo. Kisoro has a high human population density and, 92.7% of the households are involved in either crop growing or livestock farming. Ninety one percent of the district is covered by water bodies or swamps and there are two major topographic areas: southern lowlands and northern highlands, surrounded by Bwindi Impenetrable National Park and Mgahinga Gorilla National Park [32, 33].

### Study design, sample collection and laboratory tests

A quantitative observational study using stratified sampling was conducted using administrative units (sub counties, parishes and villages) as sub-groups or strata.

Stratified sampling was done in all the 14 Sub counties, and 34 parishes. From all the parishes and out of 390 villages, 361 villages were randomly selected. Due to resource constraints, 10 villages were sampled per parish. From these villages, lists were generated per village indicating households with the different species of livestock. Three households were randomly chosen per village. One animal was randomly chosen per household. Due to accessibility encumbrances, in some villages less than three households were sampled hence disproportionate sampling was done in the field (Table 1) to have a maximum of 1000 for the study. Ultimately, 338 cattle, 323 sheep and 336 goats were sampled and before blood was collected, the reproductive history and location was taken from the owner or caretaker to ascertain whether any abortion(s) had ever been experienced.

**Table 1:** RVF IgG reacting antibodies of livestock from Kisoro district, Western Region, Uganda (2016)

	Bovine	Ovine	Caprine	Total
Sub counties located in the Southern lowlands				
Nyakabande	0/20 (0.0)^a^	0/9 (0.0)	1/23 (4.3)	1/52 (1.9)
Kanaba	2/27 (7.4)	3/23 (13.0)	1/23 (4.3)	6/73 (8.2)
Murora	5/19 (26.3)	2/22 (9.1)	0/22 (0.0)	7/63(11.1)
Chahi	4/24 (6.7)	0/18 (0.0)	0/21 (0.0)	4/63(6.3)
Nyarusiza	2/40 (4.9)	0/35 (0.0)	0/10 (0.0)	2/85(2.4)
Muramba	0/24 (0.0)	0/43 (0.0)	0/10 (0.0)	0/77(0.0)
Kisoro^b^	0/5 (0.0)	0/5 (0.0)	1/88 (1.1)	1/98(1.0)
Nyakinama	2/17 (11.8)	0/51 (0.0)	0/40 (0.0)	2/108(1.9)
Sub total	15/ 176 (8.5)	5/206 (1.0)	3/237 (1.3)	23/619(3.7)
Sub Counties located in the Northern highlands				
Nyabwishenya	13/23 (56.5)	0/0 (0.0)	0/0(0.0)	13/23(56.5)
Nyundo	1/15 (6.7)	11/25 (44.0)	6/27 (22.2)	18/67(26.9)
Bukimbiri	3/24 (12.5)	4/22 (18.2)	2/22 (9.1)	9/68(13.2)
Busanza	0/20 (0.0)	1/25 (4.0)	0/23 (0.0)	1/68(1.5)
Nyarubuye	8/23 (34.8)	0/23 (0.0)	1/23 (4.3)	9/69(13.0)
Kirundo	30/60 (50.0)	1/22 (4.5)	0/4 (0.0)	31/86(36.0)
Sub Total	55/165 (33.3)	17/117 (14.5)	9/99(9.1)	81/381 (21.8)
Total	70/341(20.5)	22/323 (6.8)	12/336(3.6)	104/1000 (10.4)

^a^Positive/Total tested (%)

^b^Municipal council

Under proper restraint, 5 to 10 mL of blood was collected from the jugular vein using sterile vacutainer tubes (Becton Dickinson®, Plymouth, U.K.), labeled and allowed to clot for 30 min, and then kept at 4 °C. Within 18 h, the blood was transported from the field to the National Animal Disease Diagnostics and Epidemiology Centre (NADDEC) where serum was extracted and placed into cryogenic vials, labeled and immediately tested for IgG RVF virus antibodies following the manufacturer’s instructions using the competitive ELISA test kits:13. FVR.K.3/2 and 13. FVR.K.3/5 Ingezim Rift Valley Fever™ Compac (Immunologia y Genetica Aplicada S.A, Madrid, Spain). Serum was considered positive when ELISA reader generated an inhibition percentage (IP) ≥ 45%, tested negative with an IP ≤ 40%. Doubtful samples: 40 < IP < 45% were run again until consistent readings were obtained.

### Data management and analysis

The data was collected and analyzed using Microsoft 2010 excel (https://products.office.com/en-us/microsoft-excel-2010) using the Chi-Square test of independence.

## Results

The overall RVFV seroprevalence was 10.4% (Table 1). Cattle had the highest seroprevalence (7%), followed by sheep (2.2%) and least in goats (1.2%) (Table 2). Intra-species RVFV seroprevalence was highest in cattle (20.5%), followed by sheep (6.8%), then goats (3.6%). RVFV seroprevalence in the six sub counties located in northern highlands (21.8%) was significantly higher (*p* < 0.001) than in the eight sub counties in the south lowlands (3.7%). The overall prevalence of abortion was 17.4% and sheep had the highest (7.8%), followed by goats (6.3%) and then cattle (3.3%). Intra-species abortion prevalence was highest in sheep (24.1%), followed by goats (18.8%) and cattle (9.7%).

**Table 2:** Abortion frequency and IgG antibody seroprevalence of Rift Valley Fever in livestock species of Kisoro District, Western Region of Uganda in 2016

Species	Abortion			No Abortion			Grand Total
	RVF + ^a^	RVF -^a^	Total	RVF +	RVF -	Total	
Bovine	9 (27.3)^b^	24 (72.7)	33 (19.0)	60 (19.5)	248 (80.5)	308 (37.3)	341
Ovine	0 (0.0)	78 (100.0)	78 (44.8)	22 (9.0)	223 (91.0)	245 (29.7)	323
Caprine	4 (6.3)	59 (93.7)	63 (36.2)	9 (3.3)	264 (96.7)	273 (33.0)	336
Grand Total	13(7.5)	161 (92.5)	174 (100.0)	91 (11.0)	735 (89.0)	826 (100)	1000

^a^RVF - / + = RVF Reacting antibody negative / positive; ^b^Positive (%)

## Discussion

The purpose of this study was to determine RVF seroprevalence in the livestock species in Kisoro district, and establish whether the abortions experienced in the animals during the rainy season of “Gichurasi” could be associated with RVFV infection.

The presence of RVFV IgG reacting antibodies points to previous infection in the Kisoro district livestock population since no vaccination had ever been done before. This, coupled with optimum conditions that are similar to those in Kabale where RVFV is confirmed to be endemic, depicts that there is a high risk for amplification and spread of the RVFV within livestock and to human beings in the district of Kisoro [5, 34].

Among livestock, cattle had the highest seroprevalence (7%) and highest intra-species seroprevalence (20.5%), however they had the lowest inter and intra-species rate of abortion. Livestock census records estimate the population of livestock in Kisoro at 28,000 cattle, 10,000 goats, and 40,000 sheep [33, 35]. Also, RVF is usually less severe in cattle compared to other livestock and occurs as a subclinical infection in majority of the adult susceptible and indigenous cattle which are relatively resistant to RVF [36]. Given the fact that the cattle tend to be kept longer for production (milk and beef) and reproduction, it is expected that the number of infected cattle at a particular period will always be more compared to goat and sheep and this was also observed in Rwanda where older cattle had higher seroprevalence of RVFV [31].

Kisoro elevation is 1991 m above sea level with 91% of the district covered by water bodies or swamps. It is divided into two major topographic regions: southern lowlands and northern highlands where 92.7% of the households are involved in either crop growing and/or livestock farming. Most of the population is settled in the southern lowlands [32, 33] and hence more available land for animal rearing is found within the highlands therefore a higher livestock density. Presence of Bwindi Impenetrable national park in the northern high lands most likely offers favorable conditions for zoophilic mosquitoes to survive and proliferate and, wildlife within also acts as reservoir for RVFV [37–39]. It is highly possible that the cited risk factors significantly increased the likelihood for RVFV seroprevalence in the highlands 21.8% (*p* < 0.001) compared to the south low lands (3.7%).

The overall prevalence of abortion was 17.4% and was highest in sheep (7.8%). Intra-species abortion rate was lowest in cattle (9.7%), highest in sheep (24%) and intermediate (18.8%) in goats. Sheep with non-reactive RVFV IgG antibodies were significantly associated with abortion (Fishers exact, *p* = 0.003) which was contrary to what is in literature [40]. This contradiction from what is known may be due to the fact that majority of sheep with RVFV are severely affected compared to other livestock [36, 40]. In this study, asymptomatic sheep were screened for IgG antibodies yet these antibodies develop after acute phase of disease when severe clinical signs happen, meaning it is highly unlikely that sheep affected with Rift valley fever where case fatality is 20% and, 80 to 100% abortion is recorded would be kept long enough on the farms since it is common practice to find in Ugandan rural livestock markets and abattoirs, animals brought for sale and slaughter respectively, with history of: poor milk yield, poor weight gain, abortion, failure to conceive and animals recovering from illness. It is upon this background that probably sheep with RVFV IgG antibodies in this study were not found to be significantly associated with abortion probably because severely affected ones could have been sold off or slaughtered. Generally goats are less severely affected than sheep, with much lower morbidity and mortality [36, 40]. For cattle and goats, the abortion burden noted from the study could be due to other causes such as brucellosis. Indeed, 17% was the seroprevalence of brucellosis in livestock according to a previous study in 2015 in South Western region of Uganda and endemicity of the disease in Uganda is well documented in all the regions [41–44].

Conditions in Kisoro are favorable for RVFV amplification and spread with high likelihood for outbreaks in livestock and human beings. Kisoro borders Nyagatare located in Rwanda, Nyagatare located to the south has a seroprevalence of 7.9% in cattle. Kisoro borders Kabale in the east which is also endemic to RVFV [31, 45]. Kisoro is a conduit for animal movement between these areas (Kabale and Rwanda) hence the livestock and humans within Kisoro are at a high risk of being exposed to infection. Kisoro experiences two rainy seasons (September to December and March to May) with temperatures ranging from 10^o^c to 26^o^c and average humidity of 80%. Ninety one percent of the district is covered by water bodies or is swampy [32]. Ultimately, weather conditions for Kisoro are optimal for mosquito proliferation and RVFV amplification [5]. Presence of RVFV in Kisoro poses high risk for outbreaks in the local livestock, indigenous population and among the refugee population living in less optimum conditions within refugee camps in Kisoro. Globally presence of RVFV poses a pandemic risk since many tourists visit the national parks: Bwindi Impenetrable National Park, Mgahinga Gorilla National Park, Rwanda’s Parc National des Volcans and the Virunga National park in DRC, located within or in districts and countries neighbouring Kisoro district. The situation in Kisoro district depicted by this study is of public health importance especially in current circumstances where vaccines are not readily available to control amplification and spread of RVFV, therefore awareness through risk communication to members of the communities as shown by the study by Annabelle et al., would improve the knowledge, attitudes and perceptions about the disease and associated risk factors hence increasing the capacity of community members to prevent, detect and quickly respond to disease in livestock and zoonotic infection [34].

## Conclusion

RVF is endemic in Kisoro as shown by consistent RVFV reacting antibodies among cattle, sheep and goats. Livestock in the highland areas are more likely to be exposed to RVFV infection compared to those in the southern lowlands. Since animals move from one area of the country to another, the likelihood for RVF outbreak in any part of Uganda is high when important risk factors are present such as: above-normal cumulative rainfall that leads to an increase in mosquito breed sites; development of previously laid eggs by infected females into mosquitoes to form a critical number of vertically infected adults; exposure to infected mosquitoes and transmission after a viremia phase to others non infected mosquitoes increasing the risk of transmission to non-immune animals that are permissive to infection.

Studies that would identify the phylogeography of RVFV would be important in terms of identifying the origin of infection in Kisoro district so as to institute targeted prevention and control measures in the places of origin.

The input of the Agriculture ministry (MAAIF) in ascertaining endemicity of RVFV in the country and creating awareness that would culminate in mass vaccination of the national herd against RVF would reduce the risk of infections in animals that would spill over into the human population. Sentinel surveillance and monitoring of climatic parameters: precipitation, Sea Surface Temperatures (SST) and Normalized Difference Vegetation Index (NDVI) would aid in timely prevention, detection and response to outbreaks.

## Abbreviations

DRC: Democratic Republic of the Congo
ELISA: Enzyme-Linked Immunosorbent Assay
IgA: Iimmunoglobulin A
IgG: Immunoglobulin G
MAAIF: Ministry of Agriculture, Animal Industry and Fisheries
ml: Millilitres
NADDEC: National Animal Disease Diagnostic and Epidemiology Centre
NDVI: Normalized Difference Vegetation Index
NEMA: National Environment Management Authority
RT –PCR: Real Time-Polymerase Chain Reaction
RVF: Rift valley fever
SST: Sea surface temperatures
U.K: United Kingdom
UNCST: Uganda National Council for Science and Technology
UVRI: Uganda Virus Research Institute
Vol: Volume
